# TRPV1 is crucial for thermal homeostasis in the mouse by heat loss behaviors under warm ambient temperature

**DOI:** 10.1038/s41598-020-65703-9

**Published:** 2020-05-29

**Authors:** Park Yonghak, Seiji Miyata, Erkin Kurganov

**Affiliations:** Department of Applied Biology, Kyoto Institute of Technology, Matsugasaki, Sakyo-ku, Kyoto, 606-8585 Japan

**Keywords:** Neuroscience, Physiology

## Abstract

Thermal homeostasis in mammalians is a self-regulating process by which biological systems maintain an internal thermal stability, even under different temperature conditions; however, the molecular mechanisms involved under warm ambient temperature remain unclear. Here, we aimed to clarify functional significance of transient receptor potential vanilloid receptor 1 (TRPV1) under warm ambient temperature. TRPV1 KO mice exhibited transient hyperthermia when exposed to 30.0 and 32.5 °C, whereas wild-type (WT) mice did not. TRPV1 KO mice exhibited prolonged and prominent hyperthermia upon exposure to 35.0 °C, whereas WT mice showed transient hyperthermia. Hyperthermia also occurs in WT mice that received intracerebroventricular injection of TRPV1 antagonist AMG9810 upon exposure to 35.0 °C. Heat loss behaviors, sleeping and body licking, were deficient in TRPV1 KO mice exposed to warm temperatures. Therefore, the present results indicate that central TRPV1 is crucial for maintaining a constant body temperature via the initiation of heat loss behaviors under warm ambient temperature.

## Introduction

The thermoregulatory system of mammals maintains a relatively consistent internal temperature despite large variations in environmental conditions. Thermal homeostasis is a process that biological systems use to preserve a stable internal state for survival, which renders more robust and efficient nutrition, metabolism, and excretion and permits more precise and powerful functioning of the nervous and muscular systems^1^. Core body temperature is controlled by the central nervous system in an orchestrated manner with the help of signals from temperature-sensitive receptors expressed in peripheral nerve endings^2^ and hypothalamic regions^3^. Following environment temperature changes, various counter defense responses occur in the brain to stabilize body temperature. Previous studies suggested that core body temperature is regulated by feedback and feedforward mechanisms^4–6^. Signals on core body temperature in the brain are a feedback input, while sensory signals from peripheral nerve endings are a feedforward input. For example, an adverse increase in environmental temperature causes mammals to maintain core body temperature by releasing heat through the skin^7^. Other studies indicated that an increase in environmental temperature induced various responses including saliva spreading and hyperthermia^8^ and cold-seeking behavior^9^.

An external temperature change is initially detected by primary afferent nerve fibers of the somatosensory system at the skin. Transient receptor potential (TRP) ion channels are highly suited to function as molecular sensors for environmental stimuli^10^. Recently, STIM1 is identified to serve as thermosensor in keratinocytes to define the optimal preference temperature^11^. Transient receptor potential vanilloid receptor 1 (TRPV1), a member of the vanilloid TRP family, is a non-selective cation channel with high calcium permeability and was initially identified as a receptor for capsaicin, the pungent compound of chili pepper^12^. TRPV1 is also activated by noxious heat of more than 43 °C^12^, external pH^13^, and mechanical pain^14^. Peripheral administration of TRPV1 agonists induces hypothermia^12^. However, that of TRPV1 antagonists generates hyperthermia^15–17^, indicating that peripheral TRPV1 is continuously activated to maintain a constant thermal homeostasis^18^. Central administration of TRPV1 agonists induces transient hypothermia^19^. But, that of TRPV1 antagonists had no effect on body temperature^15,16,19,20^, indicating that TRPV1 in the brain is inactivated under normal conditions^19^.

Although TRPV1 knockout (KO) mice reveal normal responses to noxious mechanical stimuli, they are impaired in recognizing painfull heat, but only in the threshold at more than 50 °C^21^. The ablation of TRPV1-expressing somatosensory neurons, but not TRPV1 gene, impairs avoidance to extrem heat in two-plate preference tests^22^. Moreover, TRPV1 KO mice and triple KO animals of TRPV1, TRPM2, TRPM3 are dispensable for warm sensation in a goal-directed thermal perception task^23^. Although TRPV1 has been shown to be obviously involved in body temperature regulation by pharmacological studies, the evidence that TRPV1 lacks the importance for peripheral warm sensation leads to the question whether TRPV1 is really necessary for thermal homeostasis in response to warm stimulation.

To directly address this issue, the present our study aimed to elucidate whether TRPV1 is activated in naïve mice and is involved in body temperature regulation during increase of ambient temperature. In the present study, we investigated the effects of various warm environmental temperature on core body temperature, locomotor activity, and heat loss behaviors using TRPV1 KO mice and pharmacological tool. We found that hyperthermia occurred in TRPV1 KO mice but not WT animals upon warm ambient exposure. The similar hyperthermia was observed when mice received intracerebroventricular (i.c.v.) injection of TRPV1 antagonist AMG9810. Moreover, TRPV1 KO mice showed attenuated heat loss behaviors, sleeping and body licking, upon warm ambient exposure. Thus, the present results indicate that TRPV1 in the brain is important to maintain a normal safety body temperature via controlling heat loss behaviors under warm ambient temperature.

## Results

**Figure 1:**
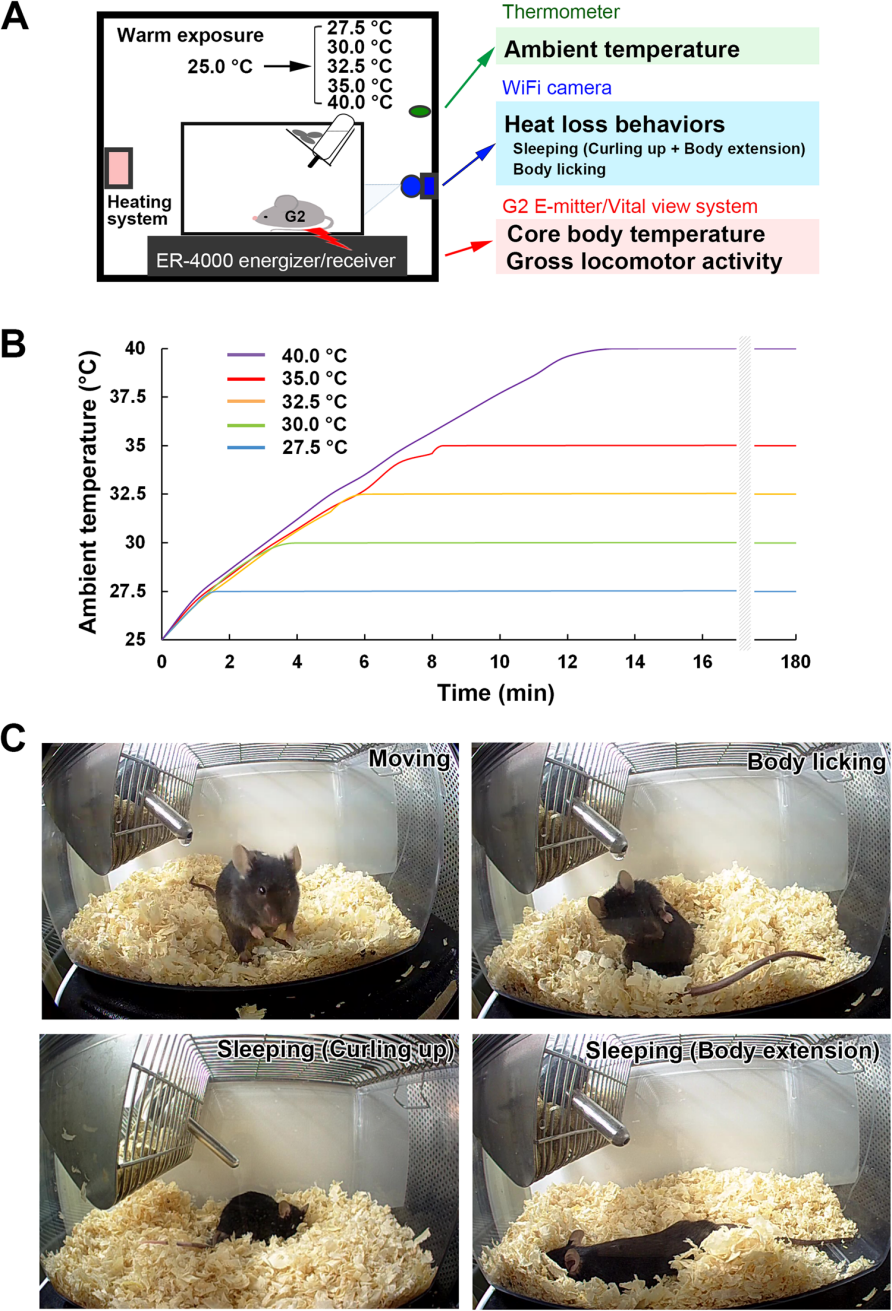
Experimental design for measuring body temperature and behaviors upon warm temperature exposure. Schematic diagram showing the methodology employed to record core body temperature and behaviors (**A**). To measure body temperature, mice were implanted with a G2 E-Mitter and kept in a clear plastic cage. The transmitter frequencies of the E-Mitter were monitored by ER-4000 receivers and transferred to Vital View software that transformed frequencies to core temperature and signal strength to locomotor activity. Heat loss behaviors, sleeping and body licking, were analyzed manually by video recorded with a WiFi camera. A representative graph shows time-course changes in the ambient temperature of the chamber (**B**). The ambient temperature was increased from 25.0 to 27.5, 30.0, 32.5, 35.0 and 40.0 °C and maintained at the given levels (**B**). Representative images reveal that mice were moving (upper left), licking their body (upper right) and sleeping (bottom left, curling up; bottom right, extending body) following exposure to 32.5 °C (**C**).

To measure the effects of warm ambient temperature on core body temperature, locomotor activity, and heat loss behaviors, wild type (WT) and TRPV1 KO mice were habituated in a chamber equipped with a temperature controller, G2 E-mitter telemetry system, and WiFi camera (Fig. 1A). Mice were allowed to move freely and had ad libitum access to food and water during measurements. The chamber temperature was raised from 25.0 to 27.5, 30.0, 32.5, 35.0 and 40.0 °C. Figure 1B shows that ambient temperature in the chamber increased from 25.0 to 27.5 °C within 1.6 min, 30.0 °C within 4 min, 32.5 °C within 6 min, 35.0 °C within 8.3 min and 40.0 °C within 12.5 min. In the behavioral analysis, the time spent sleeping and body licking was measured because these are heat loss behaviors that decrease core body temperature. Representative images show the state of active moving, body licking, and sleeping (Fig. 1C). Mammals, including humans, prepare for sleep by curling up and then fall into a deep sleep with body extension. The basal metabolic rate during sleep is lower than that during wakefulness at complete rest and in the active moving state^24,25^. Body licking or saliva spreading results in the evaporation of saliva from the surface of skin, which reduces core body temperature^7^.

**Figure 2:**
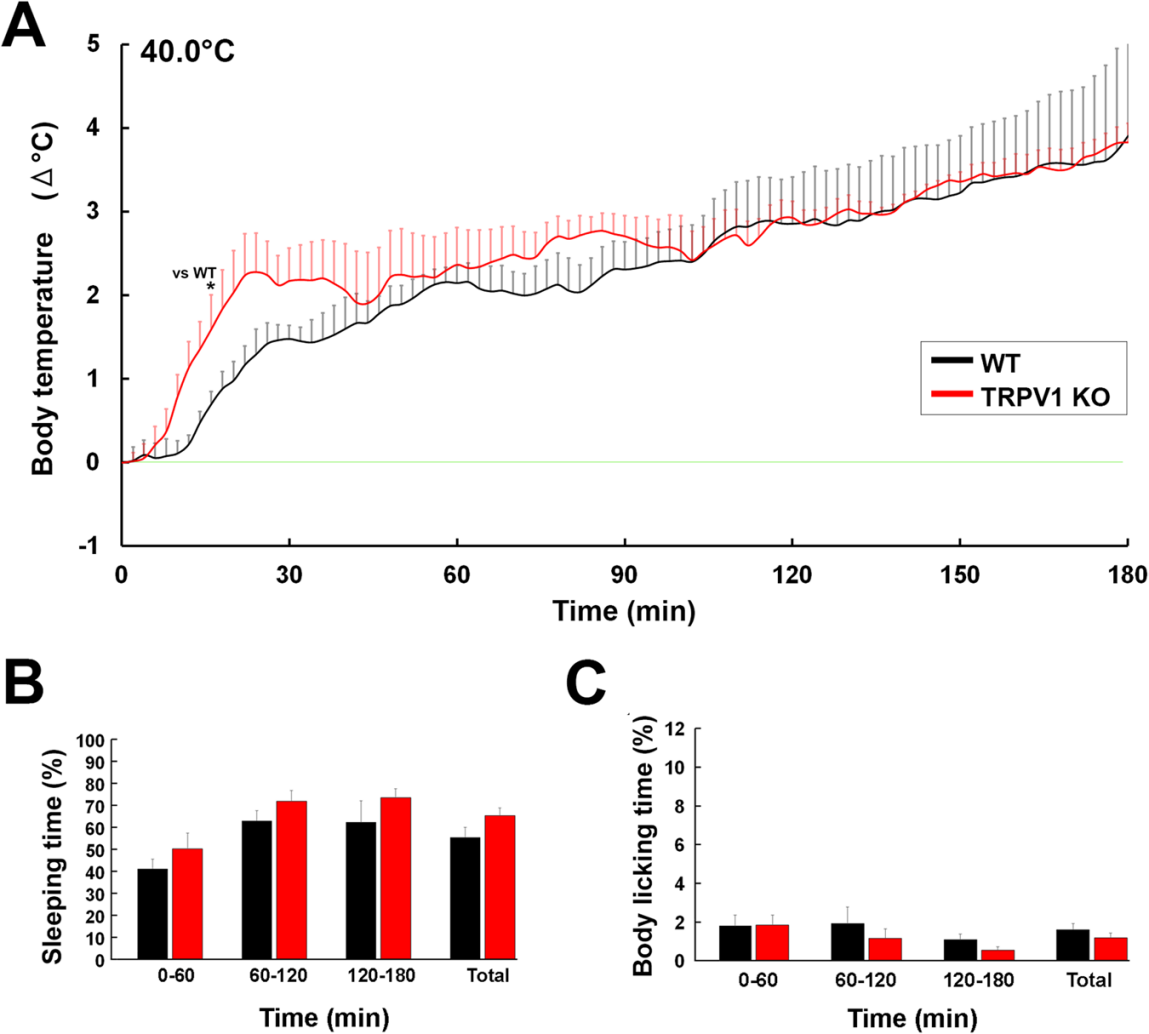
No difference of change in core body temperature between WT and TRPV1 KO mice upon heat exposure. Abdominal core temperature was measured by a G2 E-mitter telemetry system and plotted at 2-min intervals. Behaviors of mice were monitored by a WiFi camera and the time spent on heat loss behaviors, sleeping and body licking, was manually measured. The ambient temperature was increased from 25.0 to 40.0 °C and maintained at 40.0 °C. WT and TRPV1 KO mice showed almost same increase in core body temperature upon the heat exposure (**A**). There was no significant difference of sleeping (**B**) and body licking time (**C**) between WT and TRPV1 KO mice. Data (n = 5) are expressed as means (± s.e.m.). *p < 0.05 between WT and TRPV1 KO mice (Unpaired Student’s *t*-test).

To elucidate functional significance of TRPV1 in body temperature regulation, we firstly examined changes in core body temperature of WT and TRPV1 KO mice upon heat exposure at 40.0 °C (Fig. 2). Both WT and TRPV1 KO mice showed prolonged and robust hyperthermia upon exposure to 40.0 °C (Fig. 2A). Core body temperature in WT and TRPV1 KO mice quickly elevated during first 30 min and thereafter gradually increased. No significant difference was noted in core body temperature between WT and TRPV1 KO mice after the exposure, except during 14 to 16 min. There was no significant difference of the temperature index between WT (0–60 min, 1.25 ± 0.14; 60–120 min, 2.44 ± 0.36; 120–180 min, 3.37 ± 0.77) and TRPV1 KO (0–60 min, 1.76 ± 0.39; 60–120 min, 2.69 ± 0.29; 120–180 min, 3.41 ± 0.07) mice (Fig. S1). To investigate heat loss behaviors, sleeping and body licking were observed using a WiFi camera and their durations were manually counted. The durations of sleeping (Fig. 2B) and body licking (Fig. 2C) were not significantly different between WT and TRPV1 KO mice. These results were well agreement with the previous studies showing no significant difference of core body temperature between WT and TRPV1 KO mice upon heat exposure^15,26^.

**Figure 3:**
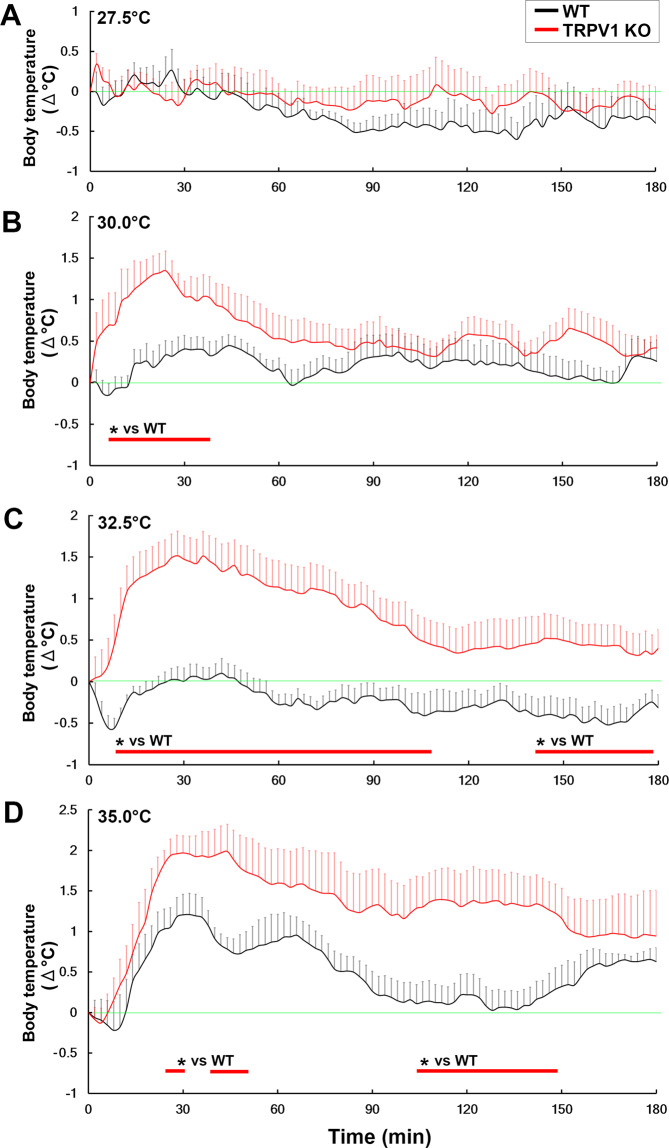
Abnormal hyperthermia in TRPV1 KO mice upon warm temperature exposure. Abdominal core temperature was measured by a G2 E-mitter telemetry system and plotted at 2-min intervals. The ambient temperature was increased from 25.0 to 27.5, 30.0, 32.5 and 35.0 °C and maintained at the given levels. WT and TRPV1 KO mice did not show significant hyperthermia upon exposure to 27.5 °C (**A**). Upon exposure to 30.0 °C, TRPV1 KO mice transiently showed mild hyperthermia, whereas hyperthermia was not observed in WT mice (**B**). Core body temperature in TRPV1 KO mice markedly increased at an early period in the exposure to 32.5 °C and then returned to initial levels in the later stages, whereas WT mice did not show any hyperthermia (**C**). WT and TRPV1 KO mice showed mild and strong hyperthermia, respectively, upon exposure to 35.0 °C, whereas the mean increase in core body temperature was significantly higher in TRPV1 KO mice than in WT mice (**D**). Although core body temperature in WT mice returned to initial levels at a later period, that in TRPV1 KO mice was continuously maintained at higher levels. The red line indicates the period in which core body temperature differed between TRPV1 KO and WT mice. The green line indicates initial body temperature. Data (n = 6–7) are expressed as means (± s.e.m.). *p < 0.05 between WT and TRPV1 KO mice (Unpaired Student’s *t*-test).

We secondly performed various warm temperature exposures of 27.5 30.0, 32.5 and 35.0 °C (Fig. 3). Core body temperature in WT and TRPV1 KO mice remained unchanged upon exposure to 27.5 °C and, thus, no significant difference was observed in core body temperature between them (Fig. 3A). Upon exposure to 30.0 °C, TRPV1 KO mice exhibited transient hyperthermia with a peak (1.34 ± 0.24 °C) at 24 min, whereas WT mice did not (Fig. 3B). The Student’s *t*-test revealed a significant difference (*p* < 0.05) in core body temperature between TRPV1 KO and WT mice from 6 to 38 min after exposure to 30.0 °C. TRPV1 KO mice showed relatively sustained hyperthermia that peaked (1.51 ± 0.29 °C) 28 min after exposure to 32.5 °C; however, no increase was observed in core body temperature in WT mice (Fig. 3C). A significant difference (*p* < 0.05) was noted in core body temperature between WT and TRPV1 KO mice from 8 to 106 min and 140 to 176 min after exposure to 32.5 °C. Upon exposure to 35.0 °C, WT and TRPV1 KO mice both showed hyperthermia that peaked (1.21 ± 0.26 °C) at 32 min and (1.98 ± 0.33 °C) at 44 min, respectively (Fig. 3D). Although WT mice showed transient hyperthermia upon exposure to 35.0 °C, TRPV1 KO mice had intense and prolonged hyperthermia. Significant differences (*p* < 0.05) were noted in core body temperature between WT and TRPV1 KO mice from 24 to 30, 38 to 48 and 104 to 146 min after exposure to 35.0 °C.

**Figure 4:**
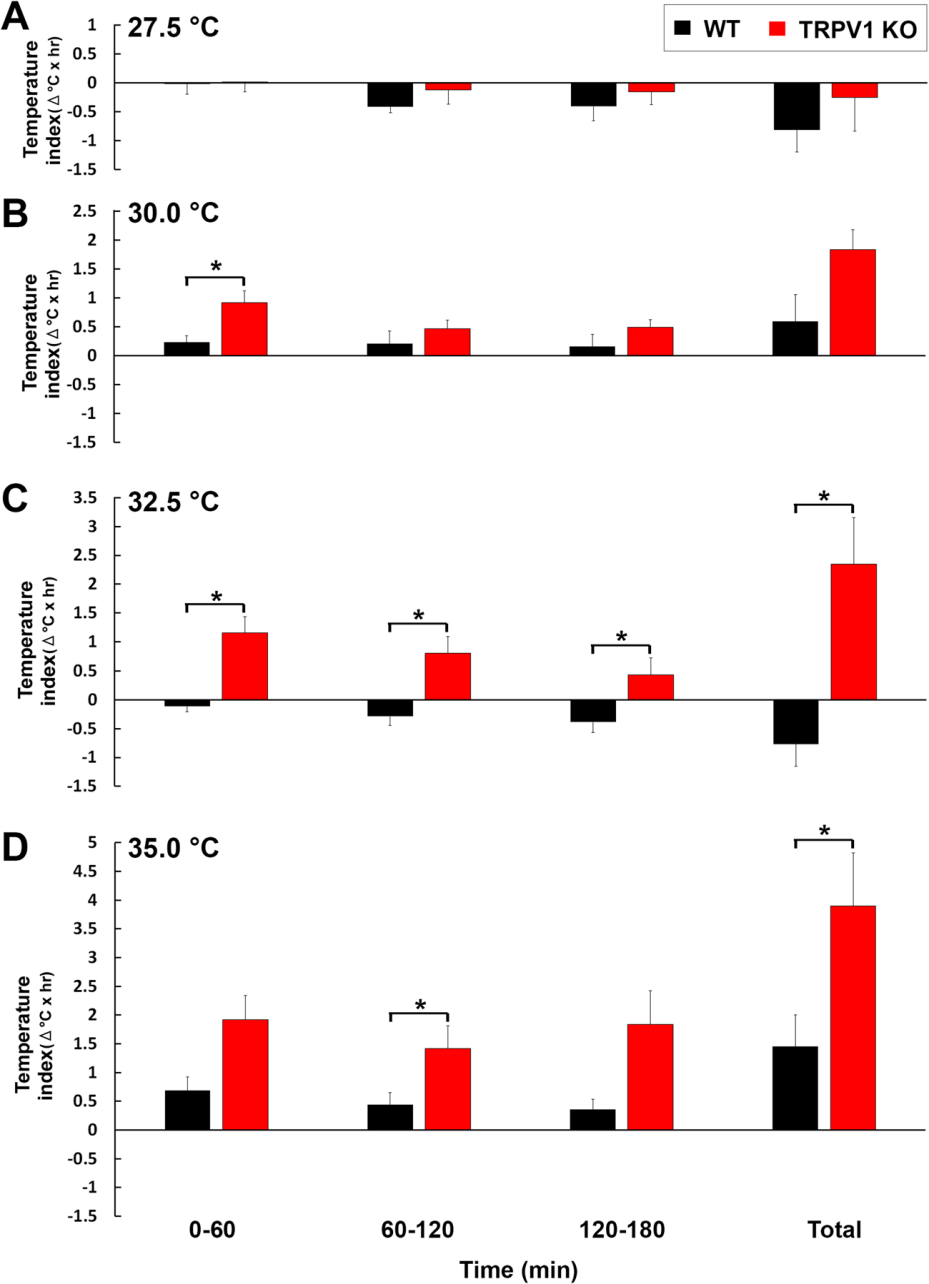
Abnormal increase in the temperature index in TRPV1 KO mice upon warm temperature exposure. Abdominal core temperature was measured by a G2 E-mitter telemetry system and the temperature index was then calculated by Δ°C × hour. The temperature index was not significantly different between WT and TRPV1 KO mice upon exposure to 27.5 °C (**A**), whereas TRPV1 KO mice showed a significantly higher temperature index during 0–60 min than that in WT mice upon exposure to 30.0 °C (**B**). The temperature index was significantly higher in TRPV1 KO mice than in WT mice in all periods upon exposure to 32.5 °C (**C**). Upon exposure to 35.0 °C, the temperature index was significantly higher in TRPV1 KO mice during 60–120 min and the total time than in WT mice (**D**). Data (n = 6–7) are expressed as means (±s.e.m.). *p < 0.05 between WT and TRPV1 KO mice (Unpaired Student’s *t*-test).

To further clarify the effects of warm temperature exposure on core body temperature in WT and TRPV1 KO mice, the temperature index was examined (Fig. 4). Neither WT nor TRPV1 KO mice showed a significant difference in the temperature index upon exposure to 27.5 °C (Fig. 4A). Upon exposure to 30.0 °C, the temperature index in TRPV1 KO mice (0.91 ± 0.21) was significantly higher (p < 0.05) than that in WT mice (0.23 ± 0.11), but only during 0–60 min (Fig. 4B). Upon exposure to 32.5 °C, the temperature index was significantly greater in TRPV1 KO mice (0–60 min, −0.11 ± 0.09; 60–120 min, −0.28 ± 0.15; 120–180 min, −0.38 ± 0.17) than in WT mice (0–60 min, 1.16 ± 0.27; 60–120 min, 0.80 ± 0.28; 120–190 min, 0.43 ± 0.28) during all periods examined (Fig. 4C). Upon exposure to 35.0 °C, TRPV1 KO mice showed a significantly higher (*p* < 0.05) temperature index during 60–120 min than WT mice (Fig. 4D). Collectively, these results demonstrated that TRPV1 KO mice lack the ability to maintain a constant body temperature upon acute exposure to warm ambient temperature.

**Figure 5:**
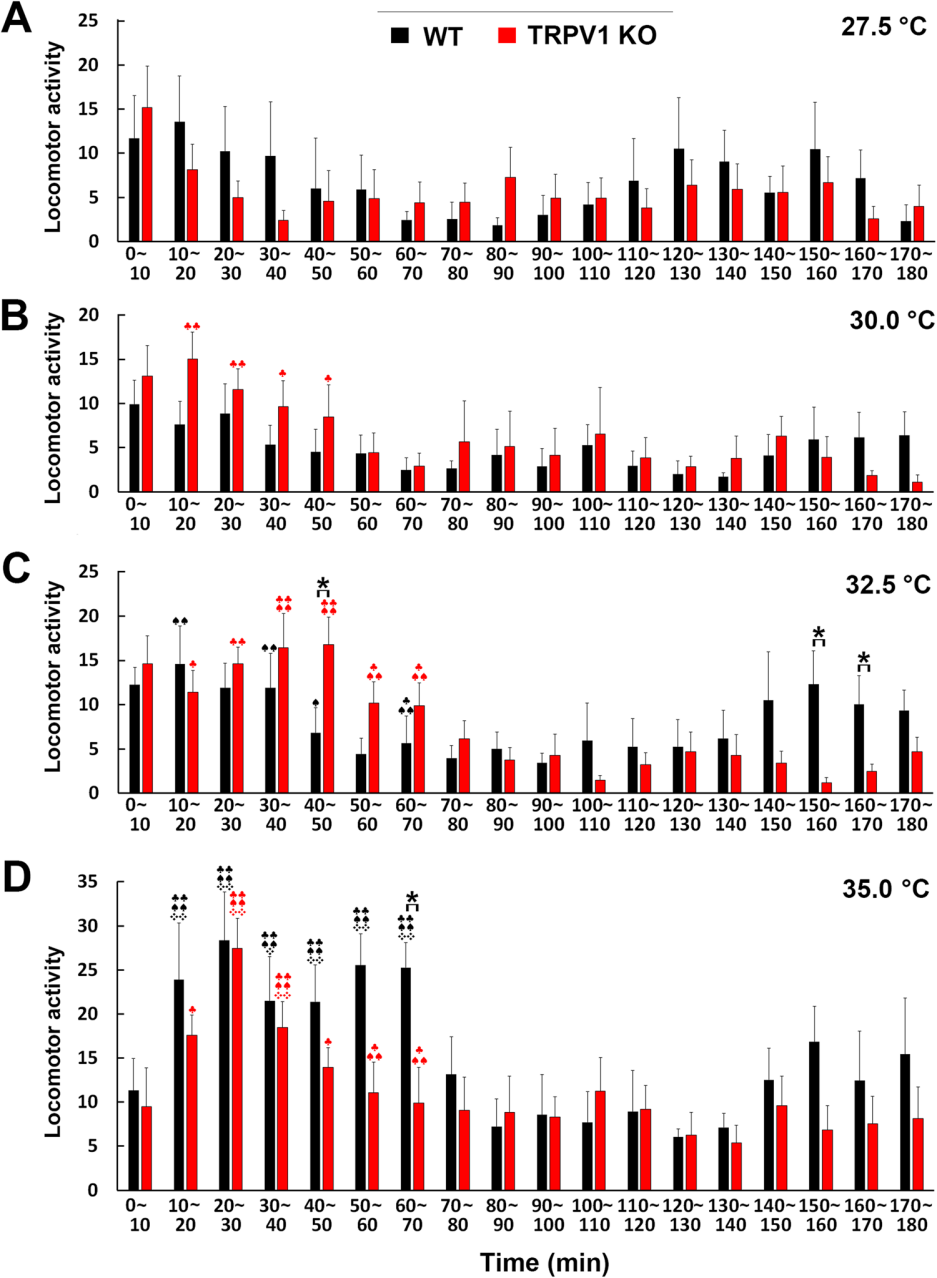
Changes in the gross locomotor activity of WT and TRPV1 KO mice upon warm temperature exposure. The mean counts of gross locomotor activity were monitored every 2 min by the G2 E-mitter telemetry system and mean gross locomotor activity was calculated every 10 min. No significant differences were observed in locomotor activity between WT and TRPV1 KO mice upon exposure to 27.5 (**A**) and 30.0 °C (**B**). TRPV1 KO mice showed slightly higher locomotor activity during the early half of the exposure to 32.5 °C than WT mice, but slightly less locomotor activity in the latter half of this warm exposure (**C**). Upon exposure to 32.5 °C, TRPV1 KO mice showed significantly higher locomotor activity during 40–50 min and lower locomotor activity during 150–170 min than WT mice. Although WT and TRPV1 KO mice both showed significantly higher locomotor activity upon exposure to 35.0 °C than to 27.5, 30.0 and 32.5 °C, WT mice were more likely to exhibit higher locomotor activity during the early and late periods of the exposure than TRPV1 KO mice (**D**). TRPV1 KO mice showed significantly lower locomotor activity during 60–70 min than WT mice. Data (n = 6–7) are expressed as means (± s.e.m.). *p < 0.05, significant difference between WT and TRPV1 KO mice (Unpaired Student’s *t*-test). ^♣^*p* < 0.05, ^♣♣^0.01, significant increase at 30.0, 32.5 and 35.0 °C vs 27.5 °C. ^♠♠^*p* < 0.01, significant increase at 32.5 and 35.0 °C vs 30.0 °C. ❖❖*p* < 0.01, significant increase between 35.0 vs 32.5 °C.

To clarify the abnormal increase in core body temperature in TRPV1 KO mice, gross locomotor activity of WT and TRPV1 KO mice was measured using the G2 E-mitter. WT and TRPV1 KO mice both showed slightly higher gross locomotor activity during the early period of exposure to 27.5 °C (Fig. 5A). TRPV1 KO mice showed higher locomotor activity at an early period of exposure to 30.0 °C than to 27.5 °C, whereas no significant difference was detected between WT and TRPV1 KO mice (Fig. 5B). WT and TRPV1 KO mice both exhibited significantly higher (*p* < 0.05) locomotor activity upon exposure to 32.5 °C than to 27.5 °C (Fig. 5C). TRPV1 KO mice exhibited significantly higher (*p* < 0.05) locomotor activity (16.77 ± 3.09) during 40–50 min than WT mice (6.82 ± 2.85) upon exposure to 32.5 °C, while locomotor activity was less in TRPV1 KO mice than in WT mice at 150–160 min (WT, 12.3 ± 3.80; KO, 1.20 ± 0.53) and 160–170 min (WT, 10.02 ± 3.22; KO, 2.48 ± 0.80). When mice were exposed to 35.0 °C, WT and TRPV1 KO mice both showed significantly higher locomotor activity during the early period than during exposure to 27.5, 30.0 and 32.5 °C (Fig. 5D). TRPV1 KO mice showed significantly lower (*p* < 0.05) locomotor activity during 60–70 min (WT, 25.26 ± 2.85; KO, 9.90 ± 4.06). These results revealed no significant differences in gross locomotor activity between WT and TRPV1 KO mice upon exposure to warm temperatures.

**Figure 6:**
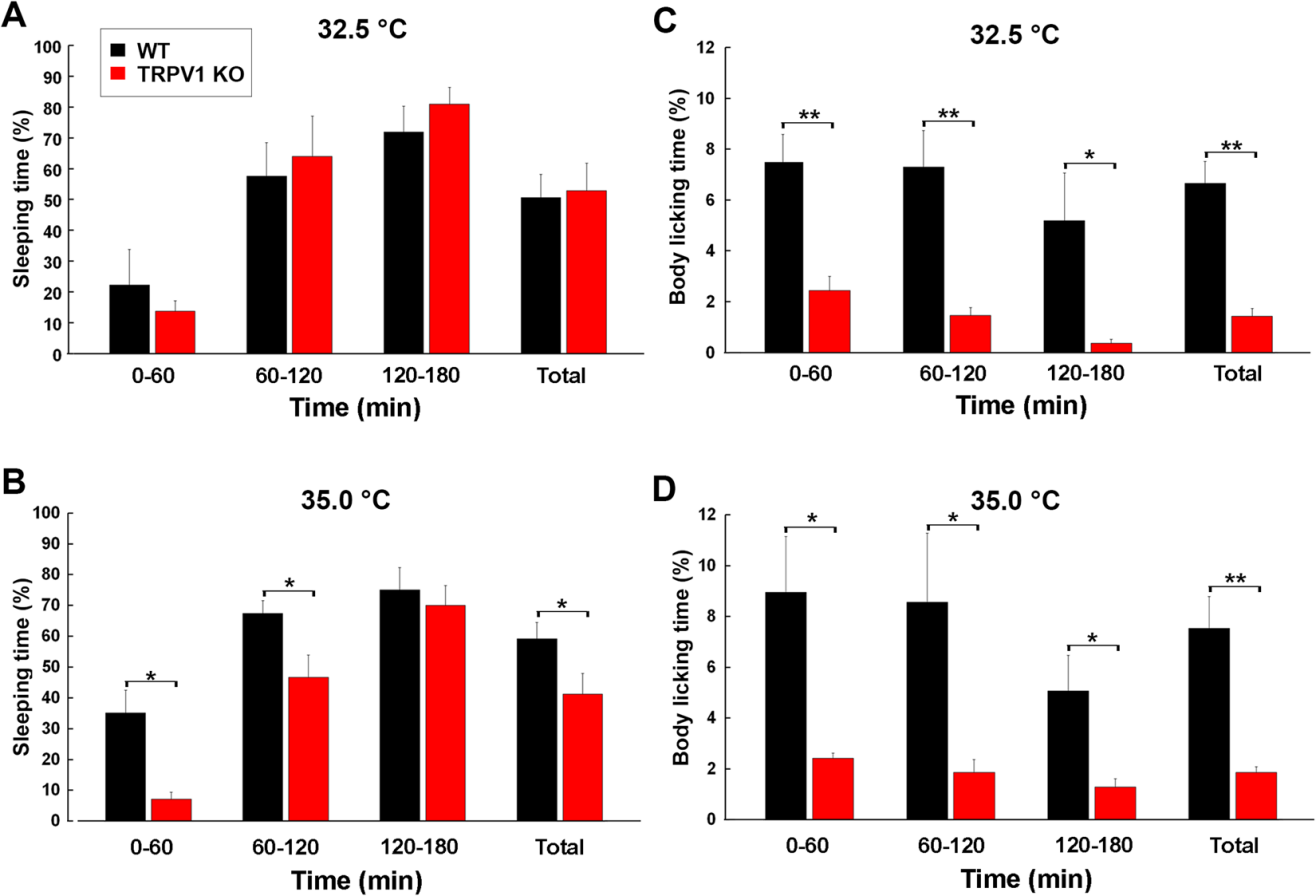
Deficient heat loss behaviors in TRPV1 KO mice upon warm temperature exposure. Mouse behaviors were recorded by a WiFi camera and the time spent on sleeping and body licking behaviors was manually counted. Sleeping time was not significantly different between WT and TRPV1 KO mice upon exposure to 32.5 ^o^C (**A**), but was significantly shorter in TRPV1 KO mice during 0–60 and 60–120 min and in the total time than in WT mice upon exposure to 35.0 °C (**B**). Body licking time was significantly shorter in TRPV1 KO mice than in WT mice upon exposure to 32.5 (**C**) and 35.0 °C (**D**). Data (n = 5–7) are expressed as means (± s.e.m.). *p < 0.05, **p < 0.01 between WT and TRPV1 KO mice (Unpaired Student’s *t*-test).

To clarify whether abnormal increases in core body temperature in TRPV1 KO mice were due to differences in their behaviors, the durations of heat loss behaviors, such as sleeping and body licking, were counted (Fig. 6). A behavioral analysis was performed at exposure to 32.5 and 35.0 °C because core body temperature markedly differed between WT and TRPV1 KO mice at these temperatures (Figs. 3–5). Body licking and extension behaviors were not observed at an ambient temperature of 25.0 °C. Upon exposure to 32.5 °C, no significant differences were observed in sleeping times between TRPV1 KO mice and WT mice (Fig. 6A); WT and TRPV1 mice both spent less time sleeping during 0–60 min, but spent more than 50% during 60–180 min. Upon exposure to 35.0 °C, sleeping time was significantly less (*p* < 0.05) in TRPV1 KO mice than in WT mice during 0–60 min (% of time: WT, 35.17 ± 7.29: KO, 7.05 ± 2.32), 120–180 min (WT, 67.38 ± 4.20; KO, 46.61 ± 7.25) and the total time (WT, 59.18 ± 5.23; KO, 41.23 ± 6.60) (Fig. 6B). Although body licking was observed in both WT and TRPV1 KO upon exposure to 32.5 °C, body licking time by TRPV1 KO mice was significantly shorter (*p* < 0.05) during 0–60 (WT, 7.48 ± 1.00; KO, 2.43 ± 0.55), 60–120 (WT, 7.30 ± 1.43; KO, 1.46 ± 0.29) and 120–180 min (WT, 5.18 ± 1.87; KO, 0.35 ± 0.15) than those by WT mice (Fig. 6C). Upon exposure to 35.0 °C, body licking time was also significantly shorter (*p* < 0.05) in TRPV1 KO mice (0–60 min, 2.42 ± 0.20; 60–120 min, 1.86 ± 0.51; 120–180 min, 1.29 ± 0.32) than in WT mice (0–60 min, 8.95 ± 2.19; 60–120 min, 8.56 ± 2.70; 120–180 min, 5.06 ± 0.41). Overall, these results demonstrated that the durations of sleeping and body licking were markedly shorter in TRPV1 KO mice than in WT mice upon exposure to warm temperatures.

**Figure 7:**
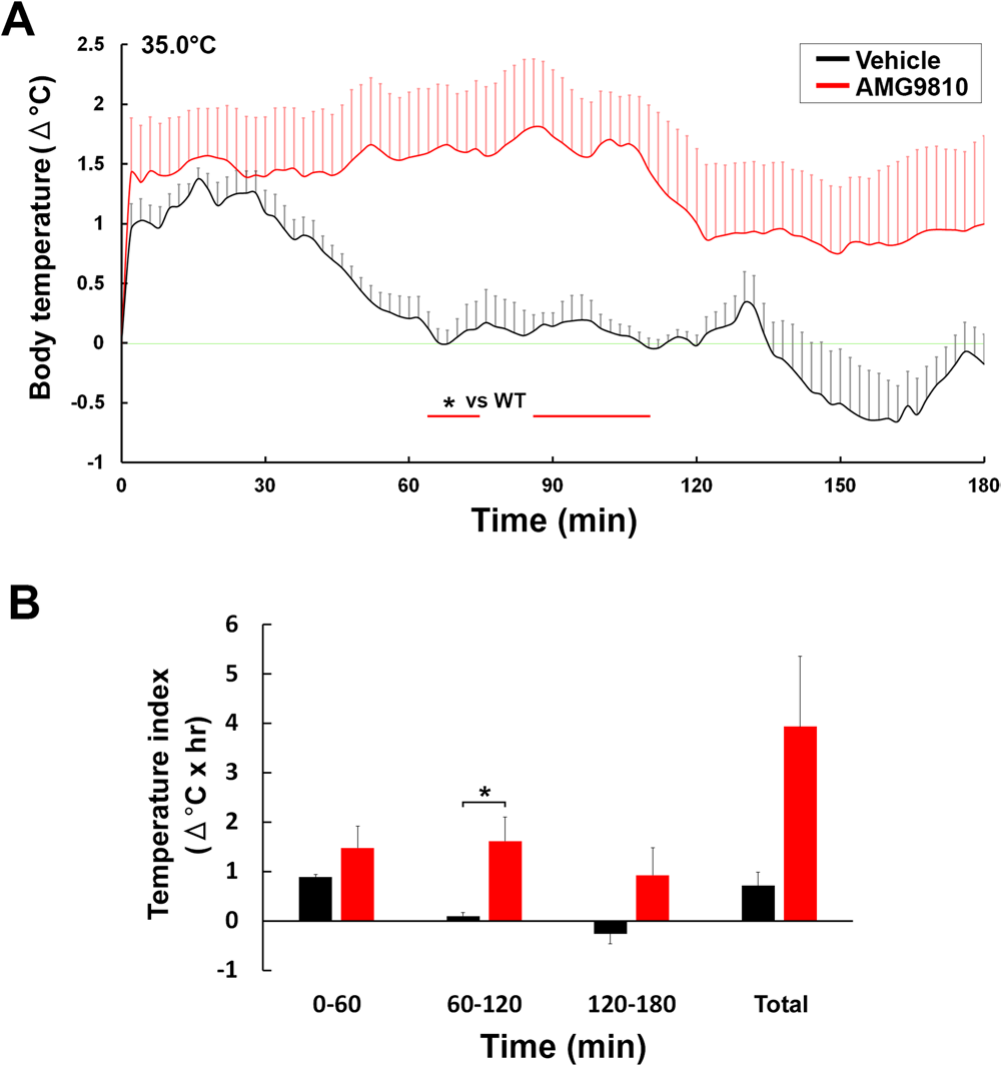
Abnormal hyperthermia in WT mice upon warm temperature exposure by i.c.v. injection of TRPV1 antagonist AMG9810. Abdominal core temperature was measured by a G2 E-mitter telemetry system and plotted at 2-min intervals. The ambient temperature was increased from 25.0 to 35.0 °C and maintained at 35.0°. Both WT and TRPV1 KO mice showed stress-induced hyperthermia just after i.c.v. injection of vehicle and AMG9810 (**A**). Core body temperature of vehicle-treated WT mice decreased to return to initial levels upon exposure to 35.0 °C, whereas that of AMG9810-treated WT animals was maintained at relatively higher levels of temperature. The temperature index was significantly higher in AMG9810-treated WT mice during 60–120 min than in vehicle-treated WT ones (**B**). The red line indicates the period in which core body temperature differed between vehicle- and AMG9810-treated WT mice. The green line indicates initial body temperature. Data (n = 5) are expressed as means (±s.e.m.). *p < 0.05 between vehicle- and AMG9810-treated WT mice (Unpaired Student’s *t*-test).

To clarify whether TRPV1 in the brain participates in controlling body temperature upon warm ambient exposure, i.c.v. injection of a TRPV1 antagonist AMG9810 was employed (Fig. 7). Core body temperature in both vehicle- and AMG9810-treated WT mice initially revealed stress-induced hyperthermia as consequence of i.c.v. injection procedure (Fig. 7A). Core body temperature of AMG9810-treated mice exhibited sustained and remarkable hyperthermia that peaked (1.76 ± 0.57 °C) at 86 min after exposure to 35.0 °C, although that of vehicle-treated WT animals decreased to the initial level within 68 min. Core body temperature of AMG9810-treated mice was likely to decrease from 108 min after the warm exposure possibly due to lowering of the antagonist. The Student’s *t*-test revealed a significant difference (*p* < 0.05) in core body temperature between vehicle- and AMG9810-treated WT mice from 64 to 72 and 86 to 108 min after the warm exposure. The temperature index in AMG9810-treated (1.65 ± 0.51) WT mice was significantly higher (*p* < 0.05) during 60–120 min than that in vehicle-treated (0.13 ± 0.09) ones (Fig. 7B). A behavioral analysis showed that the durations of sleeping and body licking were likely to be lower in AMG9810-treated WT mice than those of vehicle-treated ones, but only significantly different in total time of sleeping (Fig. S2). Collectively, these results demonstrated that inactivation of TRPV1 in the brain attenuated the ability to maintain a constant body temperature upon acute exposure to warm ambient temperature.

## Discussion

TRPV1 is a temperature-sensitive ion channel that is potentiated by temperatures higher than 42 °C in cultured cells, such as dorsal ganglion cells or TRPV1-transfected cells^12^. However, the involvement of TRPV1 in maintaining a constant body temperature under warm ambient temperature has not yet been examined *in vivo*. In the present study, we showed for the first time that TRPV1 KO mice exhibited prominent hyperthermia upon warm exposure, whereas WT mice showed no or less hyperthermia. Hyperthermia was also observed in WT mice when they received i.c.v. injection of TRPV1 antagonist AMG9810. Moreover, we revealed that TRPV1 KO animals devoted less time to heat loss behaviors, such as sleeping and body licking, than WT mice. These results indicate that TRPV1 is crucial for maintaining a normal body temperature in the adult mouse via heat loss behaviors under warm ambient temperature.

Many pharmacological studies have demonstrated that subcutaneous^15,27^, oral^28^ and central^19,29,30^ administration of TRPV1 agonists induces hypothermia. Thus, both peripheral and central TRPV1 have been well known to be concerned with thermal homeostasis by lowering body temperature. But, the evidence that TRPV1 lacks the importance for peripheral warm sensation^21–23^ rise questions what is the function of TRPV1 upon increase of acute ambient temperature. In the present study, core body temperature was significantly higher in TRPV1 KO mice than in WT mice upon exposure to 35.0 °C. Moreover, we found that significant hyperthermia only occurred in TRPV1 KO mice upon warm exposure at 30.0 and 32.5 °C, and never in WT mice. The present results largely differ from previous findings showing no significant difference in hyperthermia between WT and TRPV1 KO mice when temperature was increased to 37.0 °C^15^. Moreover, no significant difference was reported in colonic temperature between WT and TRPV1 KO mice when mice were exposed to 39.0 °C^26^. The contradiction between the present and previous studies is probably attributed to the difference of exposure temperature, because we also observed that TRPV1 KO mice revealed almost same hyperthermia to that of WT animals upon exposure to 40.0 °C. Overall, the present study is the first to show that TRPV1 is necessary for maintaining a normal body temperature under warm, but not heat ambient temperature.

Desalivated rats cannot control body temperature upon warm and hot ambient exposure and therefore body licking is important for reducing body temperature in rodents by evaporate heat loss^31–33^. Furthermore, animals extend their body to increase their surface area, which promotes the dissipation of heat^34^. Upon warm exposure, mice initially show curling up behavior, which prepares for sleep, and then fall into a deep sleep with body extension^24,25^. Specific activation of nitrergic/glutamatergic neurons in the preoptic area responsible for ambient warmth triggers both non-REM sleep and body cooling, indicating sleeping can lower body temperature and energy expenditure^35,36^. In the present study, we found prominent hyperthermia in TRPV1 KO mice, but not in WT mice exposed to 32.5 °C. Upon exposure to 32.5 °C, body licking times were shorter in TRPV1 KO mice than in WT animals, whereas no significant differences were observed in sleeping times. Upon exposure to 35.0 °C, TRPV1 KO mice exhibited stronger and longer hyperthermia than WT mice, whereas WT mice showed transient hyperthermia and their body temperature soon returned to the initial level. Under mild warm exposure conditions (32.5~35 °C), TRPV1 KO mice showed significantly shorter times for sleeping and body licking than WT mice. On the other hand, the present study showed no significant differences of sleeping and body licking times between WT and TRPV1 KO mice upon heat exposure (40.0 °C), indicating exposure to heat exposure is beyond the cooling capacity achieved by heat loss behaviors. Collectively, the present results indicate that TRPV1 is needed to attenuate hyperthermia possibly via heat loss behaviors under warm ambient temperature.

In the present study, we demonstrated that TRPV1 KO mice exhibited prominent hyperthermia and spent less time of heat loss behaviors upon warm ambient exposures. We further found that intense and prolonged hyperthermia occurred upon exposure to 35.0 °C when the TRPV1 antagonist AMG9810 was injected into the cerebroventricle of WT mice. The durations of heat loss behaviors were likely to be diminished in AMG9810-treated mice. TRPV1 is not important for peripheral warm sensation; TRPV1 KO mice reveal normal heat avoidance between 40–50 °C by two-plate preference tests^22^ and normal warm sensation between 32~42 °C by goal-directed thermal perception task^23^. Moreover, triple KO animals of TRPV1, TRPM2, TRPM3 could learn to report warming stimuli of 32~42 °C and sense small increase of warming stimuli^23^. Central injection of TRPV1 agonist is well known to induce hypothermia^19,29,30,37^. The strong expression of TRPV1 has been reported at astrocytes in the OVLT^38–40^. The OVLT sends afferent fibers directly to the preoptic area and paraventricular nucleus and indirectly to the dorsomedial hypothalamic area^41^, where are considered to be associated with body licking behavior^42^ and sleep induction^43^. Taken together with the previous studies, the present study indicates thatTRPV1 activation in the brain is necessary for thermal homeostasis and heat loss behaviors upon acute warm exposure.

The present our study indicates that TRPV1 activation in the brain is important for thermal homeostasis upon acute warm stimuli. We speculate that there are two possible mechanisms to activate TRPV1 in the brain. The first possibility is that the thermal threshold of TRPV1 in the brain is reduced by inflammatory mediators under warm ambient temperature and thereby TRPV1 acquires warm sensitivity and detects small increase in brain temperature. The threshold temperature of TRPV1 activation decreases to 35.0 °C from 42.0 °C due to the phosphorylation of TRPV1 via the protein kinase C pathway^44^. A number of inflammatory mediators have been proposed to play a role in the phosphorylation of TRPV1 and decrease its thermal threshold, such as bradykinin, cytokines, chemokines, ATP, 5-HT and prostaglandin E2^45^. Warm and heat exposure in mice induce inflammation by increasing brain levels of cytokines and prostaglandin E2-synthesizing enzyme cyclooxygenase-2^46^. Body licking behavior is caused by hypothalamic warming in the rat brain and its duration is extended as ambient temperature increases to 32.0 °C from 24.0 °C^34^. The second possibility is that TRPV1 is activated temperature-independently by endogenous ligands, such as N-arachidonoyl dopamine (NADA), lipoxygenase products of arachidonic acid and endocannabinoid^47,48^. NADA is structurally similar to capsaicin and is the most potent endogenous ligand for TRPV1 with the ability to induce cation influx via the activation of TRPV1^49^. NADA attenuates cytokine increases in the plasma of mice treated with toll-like receptor 4 agonist lipopolysaccharide and toll-like receptor 2 agonist Pam3Cys in a TRPV1-dependent manner^50^.

## Materials and methods

### Animals

Adult male C57BL/6 J strain WT and TRPV1 KO mice (70–105 days old) were housed in individually plastic cage (CL-0103–2, 182 × 260 × 128 mm; CLEA Japan Inc., Tokyo, Japan) in a colony room (25.0 ± 1 °C) with a 12-h light/dark cycle; light on at 7:00 and light off at 19:00 and given *ad libitum* access to commercial chow and tap water. TRPV1 KO mice were made by Dr. D. Julius^21^ and supplied by Dr. M. Tominaga. All experiments were performed in accordance with the Guidelines laid down by the Proper Conduct of Animal Experiments Science Council of Japan. The experimental protocol was approved by the Animal Ethics Experimental Committee of the Kyoto Institute of Technology.

### I.c.v. injection of AMG9810

AMG9810, a selective and competitive TRPV1 antagonist, inhibits capsaicin-, proton-, heat- and endogenous ligand-induced activation of TRPV1^17^. The stock solution (10 mg/ml; abcam, Cambridge, UK) was made by dissolving AMG9810 in DMSO and stored at −80 °C, and then diluted 10 times with pyrogen-free physiological saline (Otsuka Pharmaceutical Factory, Tokushima, Japan) prior to use. For icv administration, a stainless steel cannula (25-gauge) was implanted in each mouse under anesthesia with isoflurane so that its tip laid in the lateral cerebral ventricle (0.3 mm anteroposterior and 1.0 mm lateral to the bregma and 2.5 mm dorsoventral below the skull) using a standard stereotaxic technique^51^. Freely moving mice received icv administration of AMG9810 (1 mg/ml; 3 μl, 1.5 μg/kg) or 10% DMSO in pyrogen-free physiological saline using a Model EP-1000E administration pump (Melquest, Toyama, Japan; 0.5 μl/min).

### Measurement of core body and chamber temperature

Core body temperature was measured according to our previous study^19,52^. Mice were anesthetized with isoflurane, implanted intraperitoneally with a G2 E-mitter transponder (Starr Life Sciences Corp., Oakmont, PA) to record core body temperature, and then housed at an ambient temperature of 25.0 ± 1 °C under a 12-h light/dark cycle; light on at 7:00 and light off at 19:00. The G2 E-mitter is a wireless small E-mitter (dimensions 15.5 × 6.5 mm) that is useful for measuring core body temperature and gross locomotor activity in mice. Mice were kept for one week after the implantation of the transponder. The cage for mice was kept in a temperature- and light-controlled chamber with ± 0.5 °C thermal uniformity (MIR-553; interior dimensions 640 (W), 550 (D), 1,100 (H), Sanyo, Gunma, Japan). The ambient temperature was increased from 25.0 to 27.5, 30.0, 32.5, 35.0 and 40.0 °C starting at 11:00 and the chamber was maintained at a given temperature. Abdominal temperature and gross locomotor activity were measured by a biotelemetry system at 2-min intervals. The operating temperature range of the G2 E-mitter ranged between 18.0 and 42.0 °C with ± 0.1 °C thermal accuracy. The baseline temperature was obtained as the mean core body temperature in each group at 10:45–11:00. Data were acquired and fed to a computer using Vital View software (Vital View series 4000, Starr Life Science Corp). The temperature change index (Δ°C × hour) was calculated as described previously^19,40^. The ambient temperature in the chamber was monitored by a 4ch data logger thermometer (TM-947SD J, SATO Measuring Instruments, Kanagawa, Japan) and recorded by real-time aggregation software MJ-LOG2 (ATO Measuring Instruments).

### Recording of behaviors

Mice were singly housed in the cage (182 × 260 × 128 mm) and recorded with an AI WiFi camera (Yoosee Smart Camera, Guangdong, China) positioned approximately 15 cm from the side of the cage floor. The cage for mice was kept in the MIR-55 chamber with ± 0.5 °C thermal uniformity under a 12-h light/dark cycle; light on at 7:00 and light off at 19:00. The ambient temperature was increased from 25.0 to 27.5, 30.0, 32.5, 35.0 and 40.0 °C starting at 11:00 and the chamber was maintained at a given temperature. The durations of body licking and sleeping behaviors were calculated by manually analyzing recorded video. Body licking was counted when mice showed saliva spreading^53^. Sleeping behavior was counted when mice showed behaviors such as curling up or body extension^54,55^.

### Statistical analysis

All values are presented as means ± S.E. The Student’s *t*-test was used to compare group differences using STATISTICA (StatSoft Inc., Tulsa, OK). A difference was considered to be significant when *p* < 0.05.

## Supplementary information

Supplementary Information.
