# Supplementary material for: TRPV1 is crucial for thermal homeostasis in the mouse by heat loss behaviors under warm ambient temperature
Source: Sci Rep. 2020 May 29;10:8799. doi: 10.1038/s41598-020-65703-9 (PMC7260197; doi:10.1038/s41598-020-65703-9)
Supplement: Supplementary file 1 — Supplementary Information. [file 41598_2020_65703_MOESM1_ESM.pdf]

Supplementary Figures

TRPV1 is crucial for thermal homeostasis in the mouse by heat loss behaviors under warm ambient temperature

Park Yonghak, Seiji Miyata, Erkin Kurganov

Department of Applied Biology, Kyoto Institute of Technology, Matsugasaki, Sakyo-ku, Kyoto 606-8585, Japan.

Correspondence:
Dr. Erkin Kurganov, Department of Applied Biology, Kyoto Institute of Technology, Kyoto 606-8585, Japan.
Tel.: +81-75-724-7796
E-mail: kurganov@kit.ac.jp

Supplementary Figure 1

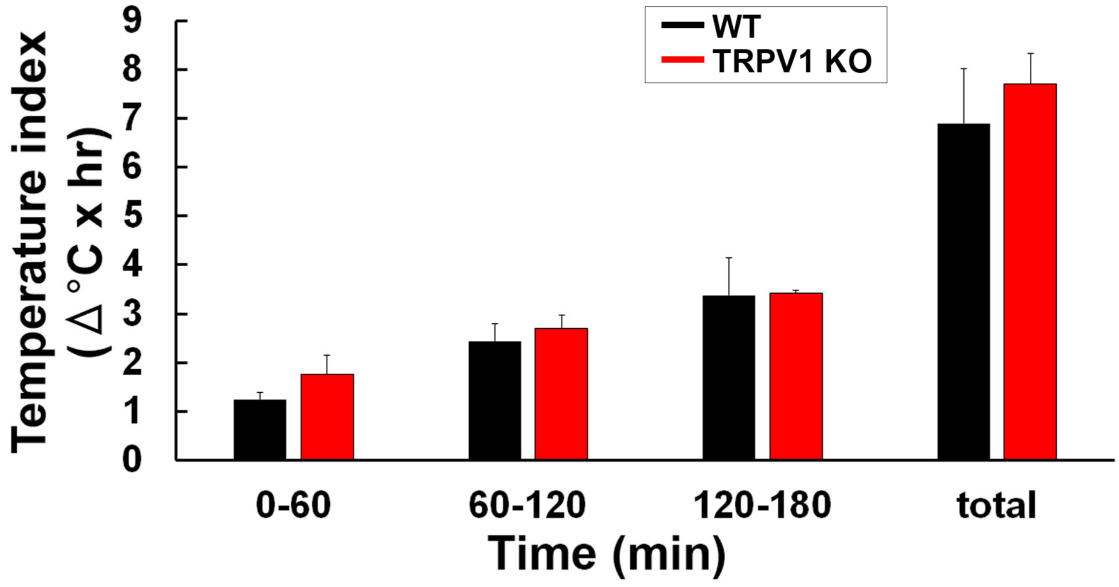

Supplementary Figure S1

No difference of temperature index between WT and TRPV1 KO mice upon heat exposure. Abdominal core temperature was measured by a G2 E-mitter telemetry system and plotted at 2-min intervals and the temperature index was then calculated by Δ°C × hour. The temperature index was not significantly different between WT and TRPV1 KO mice upon exposure to 40.0°C. Data (n=5) are expressed as means (± s.e.m.).

Supplementary Figure 2

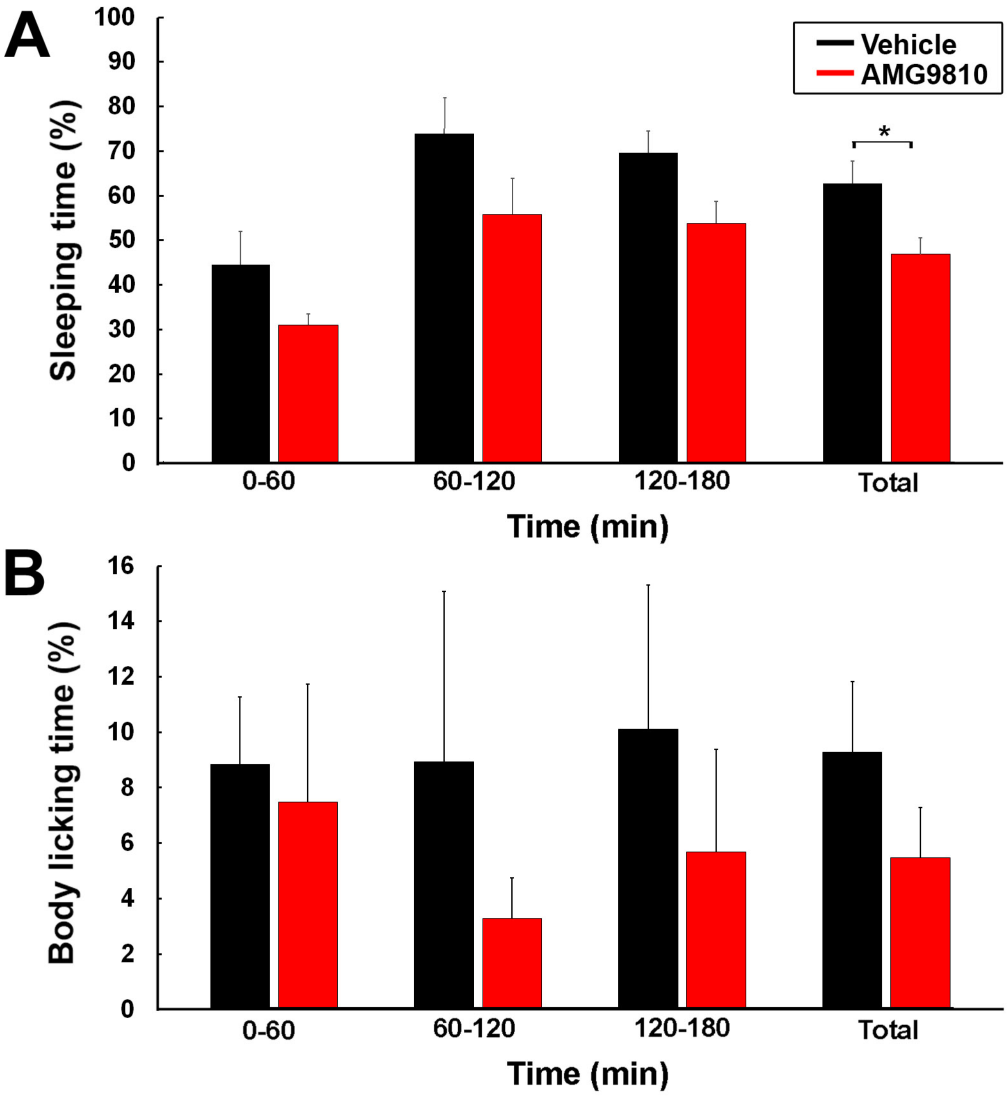

Supplementary Figure S2

Central injection of TRPV1 antagonist AMG9810 was likely to attenuate heat loss behaviors of WT mice upon warm temperature exposure. Mouse behaviors were recorded by a WiFi camera and the time spent on sleeping and body licking behaviors was manually counted. Total sleeping time was significantly shorter in AMG9810-treated WT mice than that of vehicle-treated animals upon exposure to 35.0ºC (A). Body licking time was likely to be shorter in AMG9810-treated mice than that of vehicle-treated animals (B). Data (n=5) are expressed as means (± s.e.m.). *: $p < 0.05$ between vehicle- and AMG9810-treated WT mice (Unpaired Student's *t*-test).
